# Cognitive Stimulation Interventions for Chemotherapy-Related Cognitive Impairment in Breast Cancer Patients: A Systematic Review and Meta-Analysis

**DOI:** 10.3390/cancers17183001

**Published:** 2025-09-14

**Authors:** Macarena C. Cáceres, Miguel Ángel Martín-Parrilla, Jesús Montanero-Fernández, Aitana Santos-Fernández, Casimiro Fermín López-Jurado, Noelia Durán-Gómez

**Affiliations:** 1 Departamento de Enfermería, Facultad de Medicina y Ciencias de la Salud, Universidad de Extremadura, 06005 Badajoz, Spain; mcaceres@unex.es (M.C.C.); casimirolj@unex.es (C.F.L.-J.); nduran@unex.es (N.D.-G.); 2 Grupo de Investigación Traslacional Biomédica y Sociosanitaria (CTS064), 06005 Badajoz, Spain; 3 Instituto Universitario de Investigación Biosanitario de Extremadura (INUBE), 06005 Badajoz, Spain; 4 Departamento de Enfermería, Centro Universitario de Plasencia, Universidad de Extremadura, 10600 Plasencia, Spain; 5 Departamento de Matemáticas, Facultad de Medicina y Ciencias de la Salud, Universidad de Extremadura, 06005 Badajoz, Spain; jmf@unex.es; 6 Grado en Enfermería, Facultad de Medicina y Ciencias de la Salud, Universidad de Extremadura, 06005 Badajoz, Spain

**Keywords:** breast neoplasm, chemotherapy-related cognitive impairment, cognitive training, systematic review, meta-analysis, oncology nursing

## Abstract

**Background**: A considerable proportion of breast cancer (BC) patients experience chemotherapy-related cognitive impairment (CRCI) and other symptoms even after the completion of treatment. The persistence of CRCI throughout the oncological process highlights the need for routine assessment of its severity, impact on quality of life, and the effectiveness of interventions aimed at addressing it. **Objectives**: To analyse the effectiveness of cognitive stimulation interventions on CRCI in BC patients and to identify the characteristics of such interventions, including the most appropriate timing for their implementation, the most suitable techniques, and their duration. **Methodology**: A systematic review and meta-analysis were conducted in accordance with PRISMA guidelines. Randomized controlled trials published between 1 January 2020 and 31 December 2024 were searched across three electronic databases. Studies involving cognitive stimulation interventions for the management of CRCI in BC patients were included. **Results**: A total of 12 eligible studies were identified for the systematic review and 10 for the meta-analysis. The review revealed a wide range of cognitive stimulation interventions, differing in techniques, duration, format, and timing of implementation. Group-based therapies lasting between 6 and 12 weeks predominated, with cognitive outcomes primarily assessed using the FACT-Cog scale. The meta-analysis demonstrated a moderate positive effect of cognitive stimulation interventions on cognitive functioning in BC patients (d = 0.59), although not statistically significant (*p* = 0.07), and showed high heterogeneity across studies (I^2^ = 93%). **Conclusions**: Cognitive stimulation interventions show potential benefits in improving cognitive functioning in BC patients following chemotherapy. However, the high methodological heterogeneity limits the strength of the evidence. Further research is needed to develop standardized and personalized early intervention protocols.

## Simple Summary

Cognitive difficulties are a common and often persistent side effect experienced by many breast cancer patients following chemotherapy, negatively affecting daily functioning and overall quality of life. These cognitive impairments, frequently referred to as “chemo brain”, may include problems with memory, attention, and processing speed. This study systematically reviewed and analysed recent clinical trials to evaluate the effectiveness of cognitive stimulation interventions in managing these symptoms. The review aimed to identify not only the efficacy of such interventions but also key characteristics such as optimal timing, techniques, and duration. Although the findings suggest a moderate beneficial effect on cognitive functioning, methodological variability among studies limits the strength of the conclusions. These results emphasize the need for further research to develop standardized, evidence-based approaches for early cognitive support in breast cancer care.

## 1. Introduction

Although breast cancer (BC) is one of the most prevalent cancers among women worldwide, early detection and current treatment strategies have significantly improved survival rates [1].

It has been previously demonstrated that BC patients often experience a psychoneurological symptom cluster, which includes sleep disturbances, fatigue, anxiety, depressive symptoms, and cognitive impairment [2,3,4]. These symptoms, which tend to occur concurrently during the active phase of oncological treatment, form an inseparable unit with disruptive consequences for emotional, physical, cognitive, and social functioning. Identifying symptom clusters and their relationship with clinical and therapeutic management variables is enabling a better understanding of the clinical picture of these patients and facilitating the planning of interventions to improve their quality of life (QoL) [5].

One of the primary symptoms negatively affecting the QoL in BC patients is chemotherapy-related cognitive impairment (CRCI). CRCI is characterized by cognitive deficits in areas such as memory, attention, and executive function, which can interfere with daily functioning and reduce occupational performance [6]. The prevalence of CRCI has been estimated to be as high as 75% [7], making it a growing focus of clinical and research interest. CRCI has been linked to various treatments, including radiotherapy, hormonal therapy, immunotherapy, and most notably chemotherapy [8]. Chemotherapy, in particular, has been identified as a key predictor of CRCI severity. Lower perceived cognitive functioning scores have been associated with reduced cerebral oxygenation and poorer performance on phonemic and semantic verbal fluency tasks [9].

A substantial proportion of BC patients experience CRCI even after the completion of treatment, with prevalence rates remaining elevated for more than one year, and in some cases persisting five years or more post-chemotherapy [10]. The persistence of CRCI and other symptoms throughout the disease trajectory underscores the importance of routinely assessing their severity, impact, and influence on QoL [11]. Within this context, it is equally essential to assess the effectiveness of interventions aimed at symptom improvement. Most intervention studies focus on a primary symptom and monitor whether related symptoms improve as a result of the intervention. Numerous non-pharmacological interventions have been explored for the management and prevention of CRCI. These can be broadly categorized into four main types: cognitive training or rehabilitation, cognitive behavioural therapy, physical exercise, and interventions including yoga, tai chi, or mindfulness [12].

Several meta-analyses have evaluated the effectiveness of non-pharmacological interventions in improving cognitive function in BC survivors, suggesting that such approaches should be promoted as viable strategies for preventing and treating CRCI [13,14,15].

Cognitive stimulation serves as an overarching concept that includes both cognitive training and cognitive rehabilitation, each with distinct approaches. According to Woods et al. (2012), cognitive stimulation involves group-based activities aimed at enhancing general cognitive and social functioning [16]. Cognitive training refers to repetitive, standardized exercises targeting specific domains such as memory, attention, or executive function. In contrast, cognitive rehabilitation is a personalized, goal-oriented intervention that combines cognitive training with compensatory strategies to address everyday functional challenges. This classification helps differentiate the varied approaches used in interventions for CRCI.

Although some authors have conducted systematic reviews on cognitive stimulation to address CRCI [17,18,19], to date, no review has specifically focused on the efficacy and characteristics of these therapies in BC patients. Therefore, the aim of the present study was to analyse the effectiveness of cognitive stimulation interventions for CRCI in BC patients through a systematic review and meta-analysis, and to identify key characteristics of these interventions, such as the most appropriate timing, optimal techniques, and duration.

## 2. Materials and Methods

A systematic review was conducted in accordance with the Preferred Reporting Items for Systematic Reviews and Meta-Analyses (PRISMA) statement [20]. The review was not registered.

### 2.1. Inclusion Criteria

- Studies involving women aged 18 years or older, diagnosed with stage 0 to IV BC, at any point in the treatment continuum.
- Studies investigating cognitive stimulation interventions (cognitive rehabilitation or cognitive stimulation), either as stand-alone approaches or in combination with other interventions.
- Comparators were not restricted; studies comparing the intervention to usual care, waitlist control, or other non-pharmacological interventions were eligible.
- Outcomes included at least one measure of cognitive function assessed both at baseline and at follow-up using any validated neuropsychological test, whether reported as a primary or secondary outcome.
- Among study designs, only randomized controlled trials were included.
- Pilot studies and studies published in English between 2020 and 2025 were considered.

### 2.2. Exclusion Criteria

- Studies involving patients with cancers other than BC.
- Studies that did not assess and/or report cognitive functioning as a dependent variable.
- Non-interventional studies.
- Review articles.

### 2.3. Search Strategy

A comprehensive literature search was performed on 12 January 2025, across three electronic databases: PubMed/MEDLINE, Web of Science, and Scopus.

Medical Subject Headings (MeSH) and keywords included terms related to:

- Breast cancer (e.g., “breast neoplasm” OR “breast cancer”),
- Cognitive impairment (e.g., “cognitive impairment” OR “chemotherapy-related cognitive impairment” OR “chemo brain”),
- Cognitive interventions (e.g., “cognitive stimulation” OR “cognitive rehabilitation” OR “cognitive training”).

These terms were combined using the Boolean operator AND. Relevant systematic reviews and meta-analyses were also screened, and reference lists from these reviews were examined iteratively until no further eligible studies were found.

### 2.4. Study Selection Process

All references retrieved from the electronic databases were imported into Zotero (version 7.0), a reference management software, and duplicates were removed. Following de-duplication, two independent reviewers (L.R.-S. and C.F.-M.) screened the titles and abstracts of the remaining studies for relevance. Full-text reviews were conducted for studies that met the initial inclusion criteria.

Any disagreements between reviewers during the selection process were resolved through discussion and consensus, guided by the predefined eligibility criteria. Additionally, reference lists of identified systematic reviews were manually screened to identify any further relevant studies.

**Figure 1:**
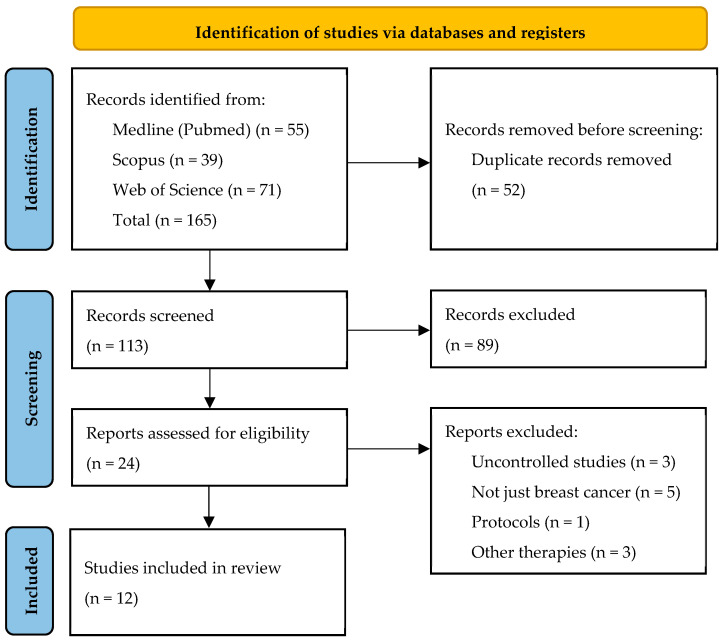
PRISMA Flow Diagram of the Study Selection Process.

A PRISMA flow diagram was used to illustrate the study selection process (Figure 1).

### 2.5. Data Extraction Process

Following the initial review of studies, a second researcher independently coded and entered information from each included study into standardized data extraction forms. Zotero software was used to manage the studies and support the data extraction process.

The extracted data included: study characteristics (first author, year of publication, regions of origin), sample size, intervention characteristics (type of intervention, intervention duration, total hours, number of sessions, setting [individual vs. group]), outcome measures used to evaluate cognitive function. Outcome data included the cognitive domains assessed and the total scores from self-reported cognitive measures, whose reliability and validity had been previously established. Additionally, data regarding the eligibility criteria, the time of CRCI measurement, and the characteristics of the control group were also extracted.

### 2.6. Risk of Bias Assessment

Two reviewers independently assessed the risk of bias for each study using the Cochrane Risk of Bias Tool version 2.0 [21]. Disagreements were resolved through discussion with the full review team.

The assessment focused on five domains:

1. Bias arising from the randomization process,
2. Bias due to deviations from intended interventions,
3. Bias due to missing outcome data,
4. Bias in the measurement of outcomes, and
5. Bias in the selection of the reported results.

### 2.7. Meta-Analysis

Data analysis was performed using IBM SPSS Statistics (version 29). A random-effects meta-analysis model for continuous outcomes was conducted. In each study, a single intervention group was compared to a control group, with cognitive outcomes assessed after the intervention.

The analysis was based on standardized mean differences (Cohen’s d) rather than raw scores, due to the heterogeneity of cognitive measures across studies. Heterogeneity was assessed using τ^2^, Higgins and Thompson’s I^2^, and Cochran’s Q test. As per Ioannidis et al. (2007), an I^2^ value above 60% was considered indicative of substantial heterogeneity [22].

Forest plots and funnel plots were used to assess heterogeneity and publication bias.

When cognitive function was assessed multiple times after the intervention, only the immediate post-intervention result was included. In studies with multiple intervention groups, these were combined into a single group using pooled means and variances.

Baseline cognitive scores were not used as the primary comparison metric due to the randomized design of all studies. Although pre-post differences would have been more informative, this approach was not feasible in all cases, as some studies did not report sufficient data to calculate the variance of the change. In two studies where baseline differences between experimental and control groups were statistically significant, post-intervention means were adjusted accordingly.

When cognitive assessment tools used inverse scoring (i.e., higher scores indicated worse performance), the direction of Cohen’s d was reversed to ensure consistency across studies.

## 3. Results

### 3.1. Intervention Characteristics

The general characteristics of the 12 studies included in this systematic review are summarized in Table 1.

A detailed description of the included studies is provided in Table 2. Multiple cognitive stimulation techniques were identified, each employing different approaches. These include: Cancer and Living Meaningfully (CALM) therapy [23,24], Brief Acceptance and Commitment Therapy (ACT) [25], Psychoeducation-based Cognitive Stimulation [26], Neurosensory Stimulation [27], Attention Process Training (APT) and Compensatory Strategy Training (CST) [28], Cognitive Behavioral Therapy for Insomnia (CBT-I) [29], Dual N-Back training [30], video game-based therapies [31,32], and cognitive stimulation combined with physical exercise or music [33,34].

Of the interventions, 58.3% (n = 7) were delivered in group settings [23,24,25,26,28,29,33], while the remainder were individual-based interventions [27,30,31,32,34].

A high degree of variability was observed in the number of sessions and overall therapy duration. Intervention lengths ranged from 2 to 24 weeks. Most studies (58.3%) were conducted over a period of 6 to 12 weeks [24,25,26,28,29,32,33]. Two studies involved shorter interventions: 2 weeks (Dual N-Back) [30] and 4 weeks (Computer-Assisted Cognitive Training [CACT]) [34]. In contrast, two studies employed longer interventions: Multisensory stimulation [27], lasting approximately 15 weeks, and video game-based cognitive stimulation [31], which extended over 6 months.

The average total duration of sessions was 15.57 h (±21.90). In eight studies (67%), the total duration was 10 h or less [23,24,25,27,29,30,33,34]. APT [28] and psychoeducation-based training [26] lasted between 12 and 15 h, while the most intensive interventions reached up to 77 h (BrainHQ and video game-based therapies) [31,32].

### 3.2. Cognitive Assessment Methods

Different eligibility criteria and cognitive assessment methods were used across studies. Some relied solely on self-reported cognitive complaints (41.7%) [26,28,30,31,32], while others used standardized cognitive scales, such as the Functional Assessment of Cancer Therapy-Cognitive Function (FACT-Cog) or Mini Mental State Examination (MMSE) [23,24,25,27,29,33,34].

The FACT-Cog was the most commonly used tool to assess CRCI, appearing in 75% of the studies [23,24,25,26,28,29,30,33,34]. This scale evaluates the impact of cognitive function on QoL, including domains such as memory, attention, concentration, thought organization, and ability to carry out daily activities.

Other instruments used included: Cognitive Failures Questionnaire (CFQ) [31], Patient-Reported Outcomes Measurement Information System (PROMIS) Cognitive Function Scale [32], Rivermead Behavioural Memory Test-Second Edition (RBMT-II) [27], and MMSE [23,24]. In general, these instruments assess domains such as memory, attention, executive function, and overall cognitive performance, either through self-report or objective testing.

### 3.3. Timing of the Intervention and Follow-Up

A subset of the selected studies (n = 5) implemented cognitive interventions during active chemotherapy treatment (defined as ≥2 chemotherapy cycles) [23,24,25,27,33]. In contrast, the remaining studies initiated cognitive training following the completion of cancer treatment, with post-treatment intervals ranging from 2 months to 5 years [26,28,29,30,32]. Notably, Smith et al. (2021) and Bellens et al. (2020) applied interventions during both treatment and the post-treatment period, thereby offering a broader scope of cognitive rehabilitation timing [31,34].

Regarding the assessment timeline, five studies conducted evaluations of CRCI both at baseline and immediately after the intervention [23,24,27,32,34]. In addition, seven studies included longitudinal follow-up evaluations ranging from 3 to 12 months post-intervention [25,26,28,29,30,31,33], allowing for a more comprehensive understanding of sustained intervention effects over time.

All included studies employed a comparative design to evaluate the efficacy of cognitive stimulation interventions. These were contrasted against control groups that received various types of alternative or minimal interventions. Importantly, five of the studies (41.6%) utilized waitlist control groups [23,24,25,26,29], providing a basis for assessing the specific effect of the active intervention in the absence of competing treatments.

Beyond the primary outcome of CRCI, several studies examined additional psychological and functional variables known to influence cognitive outcomes. These included anxiety and depression [25,28,30,31], fatigue [25], and insomnia [29]—all of which are frequently reported among cancer survivors and may exacerbate cognitive impairment. Furthermore, six studies evaluated QoL as a secondary outcome, underscoring the broader psychosocial impact of cognitive rehabilitation [23,24,30,32,33,34].

### 3.4. Risk of Bias in Studies

**Figure 2:**
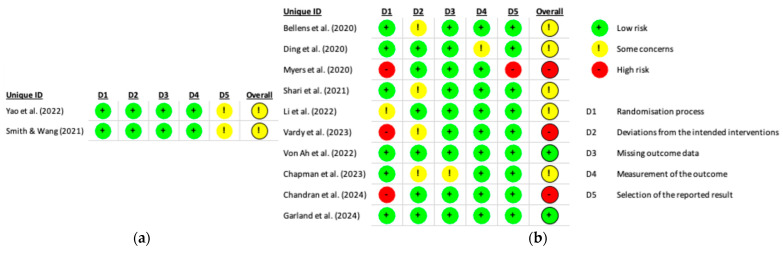
Summary of risk of bias for studies included in (**a**) the Systematic Review [24,34] and (**b**) the Systematic Review and Meta-analysis [23,25,26,27,28,29,30,31,32,33].

A detailed risk of bias assessment is presented in Figure 2.

The randomization process showed the greatest variability, with approximately 70% of studies rated as having low risk of bias, while the remainder were assessed as having either some concerns or a high risk of bias. The domain of deviations from intended interventions was predominantly rated as low risk, although around 30% of studies raised some concerns. In contrast, the domains related to missing outcome data and measurement of the outcome were associated with a high proportion, approximately 90%, of low-risk ratings. The first domain concerning the selection of the reported result revealed some concerns in 20% of studies and a high risk of bias in 10%.

Regarding the overall risk of bias, fewer than 20% of studies were judged to be at low risk. The majority were classified as raising some concerns, and approximately 25% were considered to be at high risk of bias. These findings indicate potential methodological limitations that may compromise the internal validity of the included studies.

### 3.5. Meta-Analysis

Two studies [24,34] were excluded from the meta-analysis due to insufficient data required to compute effect sizes.

#### 3.5.1. Overall Effect of Cognitive Training Interventions

**Figure 3:**
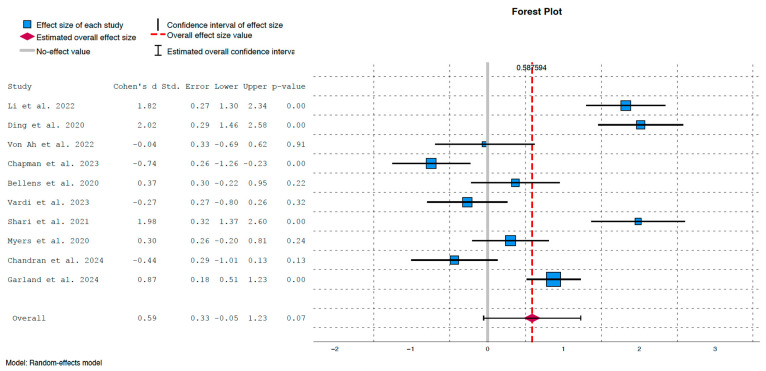
Forest plot illustrating effect sizes and confidence intervals for each individual study [23,25,26,27,28,29,30,31,32,33].

**Figure 4:**
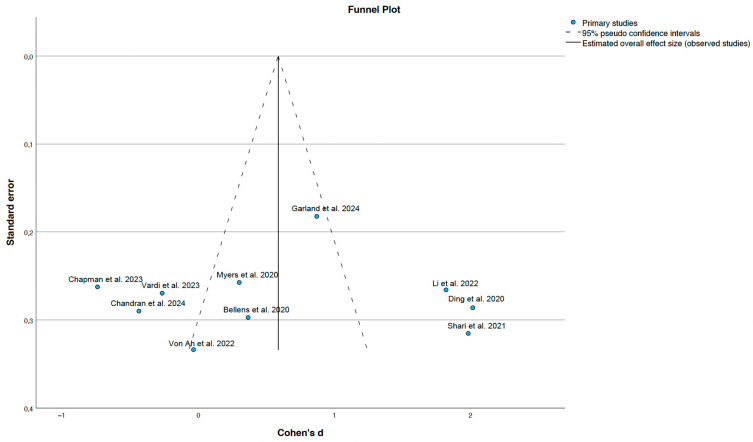
Funnel plot of standard errors and effect sizes [23,25,26,27,28,29,30,31,32,33].

The outcomes of the cognitive assessment studies are summarised in Figure 3 and Figure 4. The overall standardised mean difference between experimental and control groups was d = 0.59, with a 95% confidence interval of [−0.05, 1.23] and a *p*-value of 0.07. While this reflects a moderate positive effect of cognitive training, the difference did not reach statistical significance.

#### 3.5.2. Heterogeneity Analysis

The lack of statistical significance may be attributed to substantial heterogeneity, indicated by τ^2^ = 0.99, *p* < 0.01, and an I^2^ value of 93%, which implies that a large proportion of the variance is due to heterogeneity rather than chance. This heterogeneity is clearly depicted in Figure 3, where some studies showed strong and statistically significant effects in favour of the intervention group:

- Ding et al. (2020) [23]: d = 2.02, *p* < 0.01
- Shari et al. (2021) [25]: d = 1.98, *p* < 0.01
- Li et al. (2022) [27]: d = 1.82, *p* < 0.01
- Garland et al. (2024) [29]: d = 0.87, *p* < 0.01
- Conversely, weaker and non-significant positive effects were observed in:
- Bellens et al. (2020) [31]: d = 0.37, *p* = 0.22
- Myers et al. (2020) [26]: d = 0.30, *p* = 0.24

Moreover, several studies showed non-significant effects favouring the control group:

- Von Ah et al. (2022) [32]: d = −0.04, *p* = 0.91
- Vardy et al. (2023) [28]: d = −0.27, *p* = 0.32
- Chandran et al. (2024) [33]: d = −0.44, *p* = 0.13

Interestingly, the study by Chapman et al. (2023) showed a moderate and significant effect in favour of the control group immediately post-intervention (d = −0.74, *p* < 0.01) [30]. However, if we apply a Bonferroni correction due to the multiplicity of contrasts, the confidence intervals in Figure 3 are distributed as follows: (1.09, 2.54) for Li et al. (2020) [27], (1.24, 2.80) for Ding et al. (2020) [23], (−0.95, 0.87) for Von Ah et al. (2022) [32], (−1,47, 0.00) for Chapman et al. (2023) [30], (−0.438, 1.182) for Bellens et al. (2020) [31], (−1.01, 0.47) for Vardy et al. (2023) [28], (1.11, 2.85) for Shari et al. (2021) [25], (−0.42, 1.02) for Myers et al. (2020) [26], (−1.24, 0.36) for Chandran et al. (2024) [33] and (0.37, 1.37) for Garland et al. (2024) [29]. Therefore, the conclusions remain the same in all studies except Chapman et al. (2023), in which the effect in favour of control group becomes borderline [30].

#### 3.5.3. Publication Bias

The funnel plot presented in Figure 4 does not reveal any substantial asymmetry, suggesting the absence of publication bias. However, the ability to detect such bias may be limited due to the relatively uniform standard errors across the included studies. Overall, no clear evidence of publication bias was identified.

## 4. Discussion

### 4.1. Study Characteristics

This study investigated the effects of cognitive stimulation for managing CRCI in BC patients through a systematic review and meta-analysis. Out of the 165 studies initially identified, a total of 12 met the inclusion and exclusion criteria and were selected for systematic review. Nearly 80% of the included studies were conducted in North America or Asia, with minimal representation from Europe.

Cognitive stimulation therapies have long been used to support patients with dementia at various stages and are now being explored as targeted interventions for CRCI across different types of cancer. Fernandes et al. (2019) reviewed 19 studies—mostly randomized clinical trials—implementing cognitive rehabilitation programs, including structured cognitive training and compensatory strategies, in patients with non-central nervous system (non-CNS) cancers [18]. They reported significant improvements in memory, processing speed, and executive functioning, though they also noted considerable methodological heterogeneity across studies. Binarelli et al. (2021) focused on 20 computer-based interventions, primarily digital cognitive stimulation, and found positive outcomes in most studies, particularly in attention and memory domains, although the overall quality of evidence was moderate to low [19]. Iulio et al. (2019) conducted a broader review on neuropsychological impairments in non-CNS cancers, documenting frequent objective cognitive decline even in the absence of direct brain involvement [17]. They highlighted the clinical potential of cognitive rehabilitation interventions, which remain insufficiently standardized in routine care. Taken together, these reviews support the use of cognitive stimulation strategies across cancer populations while underscoring the need for more rigorous and cancer-specific studies—particularly in BC.

In addition to reviews specifically focused on cognitive stimulation, others have addressed a wider range of non-pharmacological interventions for CRCI, including cognitive stimulation, rehabilitation, and training. These reviews differ in population focus, intervention types, and the cognitive domains assessed. Four notable reviews [14,15,35,36] concentrated specifically on women with BC following chemotherapy. Floyd et al. identified limited evidence across ten randomized trials and highlighted the need for high-quality, multidomain studies [35]. Liu et al., through a network meta-analysis of 12 studies, found that psychotherapy was the most effective intervention for subjective cognition, whereas exercise and qigong yielded better outcomes on objective measures [14]. Park et al. included 23 studies (17 in quantitative synthesis) and reported positive effects of cognitive rehabilitation, cognitive-behavioral therapy (CBT), and physical activity on attention, immediate memory, and executive function. Pan Yang et al., in the most comprehensive review to date (42 studies), concluded that psychological interventions were most effective for subjective symptoms, while cognitive training and cognitive rehabilitation were more effective for working memory, learning, and executive functions [36]. Other reviews focusing on various cancer types, such as those by Mackenzie et al. and Zeng et al., found similar benefits from CBT, exercise, meditation, and cognitive training, although with greater heterogeneity and reduced applicability to BC specifically. Collectively, these reviews affirm the potential value of non-pharmacological interventions in mitigating CRCI but also emphasize the need for methodologically robust studies targeting well-defined clinical populations [12,37].

Our review observed substantial diversity in cognitive stimulation and training interventions, with durations ranging from less than 6 to more than 12 weeks, total hours from fewer than 3 to over 75, and implementation at various points in the treatment timeline (during chemotherapy or 3 months to 1 year post-treatment). The timing of intervention is a particularly important factor. The literature remains divided on the optimal period for addressing CRCI. Some authors advocate intervening during chemotherapy to mitigate its impact on QoL and support emotional and occupational recovery. However, most studies are conducted at least 6 months after chemotherapy, largely due to recruitment challenges during active treatment, when patients experience significant physical and emotional side effects and may be less willing to participate. Additionally, maladaptive coping strategies often develop during this phase, potentially compromising study outcomes [9].

When interpreting these findings, it is also important to consider patient-related factors. Root et al. (2023) have shown that advanced age, particularly in patients over 75 years, may contribute to cognitive decline through both cancer treatment and age-related processes [38]. Emerging evidence further indicates that biologic subtype, such as HER2 status, may also influence cognitive outcomes in BC patients [39]. In addition, while our review focused primarily on mean-based measures of cognitive performance, growing attention has been directed toward cognitive intra-individual variability (IIV) as a complementary and potentially more sensitive indicator of cognitive functioning in breast cancer survivors. IIV, which reflects fluctuations in performance across tasks or within repeated trials, has been proposed as an early marker of cognitive inefficiency and neurological vulnerability. A recent systematic review by Vance et al. (2025) synthesized evidence on IIV in this population, concluding that higher IIV may be associated with CRCI and could provide insights beyond traditional mean-based scores [40]. Future studies should therefore incorporate both demographic and tumor-related factors, as well as IIV measures, to provide a more comprehensive understanding of cognitive health in breast cancer survivors.

The quality assessment of the selected studies revealed considerable methodological variability. Fewer than 20% of the studies were judged to be at low risk of bias, while the majority raised some concerns, and approximately 25% were categorized as having a high risk of bias. Overall, the evidence base of this review presents a moderate risk of bias, with notable weaknesses particularly in the domains of randomization and selective reporting. These limitations may affect the internal validity of the findings and should be taken into account when interpreting the results of the systematic review and meta-analysis.

### 4.2. Intervention Effects

The results of the meta-analysis examining the effects of cognitive stimulation interventions indicate a moderate overall effect size (Cohen’s *d* = 0.59), suggesting potential benefits for cognitive functioning in BC patients experiencing CRCI. However, this effect did not reach statistical significance (*p* = 0.07), and the wide confidence interval (95% CI: −0.05 to 1.23) warrants caution in interpreting the magnitude of the benefit. Although the observed effect trends positively, the high degree of between-study variability may partly explain the lack of significance.

The heterogeneity analysis revealed substantial inconsistency across studies (τ^2^ = 0.99, *p* < 0.01; I^2^ = 93%), indicating that most of the variance in effect sizes is attributable to differences in study characteristics rather than random error. This is clearly illustrated in the forest plot (Figure 3), where some interventions [23,25,27,29] demonstrated strong and statistically significant improvements in cognitive outcomes. In contrast, other studies reported weak effects favouring the control group [28,30], with one study showing statistical significance; however, this significance was borderline when adjusted using the Bonferroni correction [30]. These discrepancies may stem from methodological differences, including the type, timing, duration, and delivery format of cognitive stimulation, as well as variations in outcome measures (e.g., FACT-Cog vs. alternative cognitive scales).

Despite the substantial heterogeneity, the direction of the effect generally favoured cognitive stimulation interventions. Moreover, the symmetrical distribution of studies in the funnel plot (Figure 4) suggests a low risk of publication bias. Nonetheless, the relatively small number of studies and the homogeneity in standard errors may limit the sensitivity of funnel plot-based asymmetry detection.

### 4.3. Limitations

This review provides a meaningful contribution to the current understanding of cognitive stimulation interventions for CRCI in BC patients; however, several limitations should be acknowledged. First, the relatively small number of included studies, combined with the restriction of the inclusion period to 2020–2025, may reduce the representativeness and generalizability of the findings. This temporal restriction was intentional, as our aim was to synthesize the most methodologically up-to-date evidence. Second, there was substantial inconsistency in how cognitive stimulation was defined and implemented, reflecting both heterogeneity in the literature and the evolving nature of these interventions. Outcome measures also varied, with some studies employing the FACT-Cog scale and others using alternative instruments, thereby complicating cross-study comparisons. Nonetheless, this variation reflects a growing methodological interest in the field. Additionally, two studies were excluded from the meta-analysis due to insufficient statistical reporting, which may have influenced the overall pooled estimate. Despite these limitations, the review offers a timely synthesis of recent evidence and identifies key directions for future research and clinical practice.

## 5. Conclusions

Cognitive stimulation and training interventions have the potential to improve cognitive functioning in BC patients who have undergone chemotherapy. However, substantial heterogeneity exists across studies in terms of both intervention characteristics and the assessment criteria used for CRCI. Consequently, further research is needed to comprehensively investigate the pathophysiology of CRCI, as well as effective methods for its prevention and treatment. Such efforts would support the development of unified strategies or early, personalised intervention protocols tailored to the specific needs of each patient.

## Figures and Tables

**Table 1:** General Characteristics of the 12 Studies Included in the Systematic Review.

Characteristic	Categories	N (%)
Region *	North America	6 (42.9)
	Europe	2 (14.3)
	Asia	5 (35.7)
	Australia	1 (7.1)
Sample Size	<50	3 (25.0)
	50–100	8 (66.7)
	>100	1 (8.3)
Intervention Setting	Individual	5 (41.6)
	Group	7 (58.3)
Intervention Duration (weeks)	<6	2 (16.7)
	6–12	7 (58.3)
	>12	3 (25.0)
Total Hours	Mean = 15.57 (21.90)	
Number of Sessions	Mean = 28.92 (29.58)	

* The total number of regions exceeds 12 because two studies were conducted across two of them.

**Table 2:** Detailed description of the studies included in the systematic review.

First Author and Year	Intervention	Sessions	Eligibility Method	CRCI Measurement	Control Group	Time of Intervention	Time of CRCI Measurement	Other Variables	Conclusions
Bellens 2020 [31]	Cognitive stimulation based on video games	60 min, three times per week, over 6 months	Self-reported cognitive complaint	CFQ, battery of questions	Standard supportive care	Not specified	Before the intervention, and at 3 and 6 months post-intervention	Anxiety and depression	Cognitive functioning improved over time
Ding 2020 [23]	CALM	3 to 6 sessions, each 30 min, over a period of 3 to 6 months	MMSE < 27 Karnofsky ≥ 80	FACT-Cog, MMSE, Fact-B	Waitlist control	6 or more chemotherapy cycles	Before and after the intervention	Psychologica distress, QoL	Positive effects on CRCI, QoL, and psychological distress
Myers 2020 [26]	Psychoeducation-based cognitive stimulation	6 sessions, 2.5 h each, over 6 weeks	Self-reported cognitive complaint and presence of perceived cognitive fatigue (PCF)	FACT-Cog	Waitlist control	Between 2 months and several years after chemotherapy	After the intervention, and at 1 month, 3 months, and 12 months follow-up	Loneliness	Preliminary evidence of efficacy as a treatment for CRCI
Shari 2020 [25]	ACT	4 one-hour sessions over 9 to 12 weeks	FACT-Cog	FACT-Cog	Waitlist control	During active treatment	Before, after the intervention, and at 3 months follow-up	Depression, anxiety, fatigue, psychological inflexibility	Effective intervention for improving subjective CRCI, anxiety, depression, and psychological inflexibility
Smith 2021 [34]	CACT	5 sessions per week for 1 month, 30 min each	Self-reported cognitive complaint	FACT-Cog	CACT with music	When patients report CRCI	Before and after the intervention	QoL, working memory	Enhanced cognitive performance
Li 2022 [27]	Multisensorial Stimulation	Four 26-day cycles (5 days in hospital, 21 at home), 20 min daily	Score < 26 on the MoCA. Completion of at least 2 out of 6 prescribed chemotherapy cycles	RBMT-II, BADS	Audiovisual stimulation	More than 2 chemotherapy cycles	Before and after the intervention		CRCI and chemotherapy-induced cognitive dysfunction were reduced
Vardy 2022 [28]	APT and CST	2 h per week for 6 weeks	“Do you feel your brain is functioning the same as before your cancer diagnosis?”	FACT-Cog	Current standard care	3 months after chemotherapy	Before, after the intervention, and at 12 months follow-up	Anxiety and depression	Improved CRCI
Von Ah 2022 [32]	BrainHQ	40 h over 10 weeks	Self-reported cognitive complaint	PROMIS Cognitive Abilities and Cognitive Concerns	Online computer-based activities	6 months after chemotherapy	Before and after the intervention	Plasma BDNF levels, QoL, and WAI	Intervention was acceptable and satisfactory for managing CRCI
Yao 2022 [24]	CALM	6 sessions, 30 min each, over 12 weeks	MMSE < 25	FACT-Cog, MMSE, Fact-B	Waitlist control	6 or more chemotherapy cycles	Before and after the intervention	QoL, systemic inflammatory markers	CRCI alleviated by reducing systemic inflammation levels
Chapman 2023 [30]	Dual n-back	12 sessions, 30 min each, over 2 weeks	Self-reported cognitive complaint	FACT-Cog and RRS	Dual 1-back training	Between 6 and 60 months after chemotherapy	Before the intervention, and at 2 weeks, 6 months, and 1 year post-intervention	CDT, anxiety, depression, QoL, EEG	Improved cognitive efficiency, working memory, inhibitory control, and cortical potentials (PCA)
Chandran 2024 [33]	MDR	2.5 h total, over 12 weeks	Undergoing active treatment	FACT-Cog	Unsupervised physical rehabilitation (home-based exercises)	During active treatment	Before, after the intervention, and at 6 months follow-up	QoL and ADLs	Effective intervention for patients experiencing CRCI
Garland 2024 [29]	CBT-I	7 sessions, 50 min each, over 7 weeks	FACT-Cog	FACT-Cog and ISI	Waitlist control	6 months after chemotherapy	Before and after the intervention, and at 3 and 6 months follow-up	Insomnia	Insomnia treatment contributed to improvements in CRCI

Note: All abbreviations are listed in the Abbreviations section.

## Data Availability

No new data were created or analysed in this study. Data sharing is not applicable to this article.

## Abbreviations

The following abbreviations are used in this manuscript:

ACT	Acceptance and Commitment Therapy
ADLs	Activities of Daily Living
APT	Attention Process Training
BC	Breast Cancer
BDNF	Brain-Derived Neurotrophic Factor
BADS	Behavioural Assessment of the Dysexecutive Syndrome
CACT	Computer-Assisted Cognitive Training
CALM	Cancer and Living Meaningfully
CBT-I	Cognitive Behavioral Therapy for Insomnia
CDT	Cognitive Dysfunction Test
CFQ	Cognitive Failures Questionnaire
CRCI	Chemotherapy-Related Cognitive Impairment
CST	Compensatory Strategy Training
EEG	Electroencephalogram
FACT-B	Functional Assessment of Cancer Therapy-Breast
FACT-Cog	Functional Assessment of Cancer Therapy-Cognitive Function
IIV	Intra-individual Variability
ISI	Insomnia Severity Index
MMSE	Mini-Mental State Examination
MoCA	Montreal Cognitive Assessment
MDR	Multidimensional Rehabilitation Program
PRISMA	Preferred Reporting Items for Systematic Reviews and Meta-Analyses
PROMIS	Patient-Reported Outcomes Measurement Information System
QoL	Quality of Life
RBMT-II	Rivermead Behavioural Memory Test-Second Edition
RRS	Ruminative Response Scale
WAI	Working Alliance Inventory
